# Incidence of Mortality among Under-Five-Year-Old Children Born to Women Living with HIV and Those Born to Women Not Living with HIV in Botswana: A 5-Year Retrospective Study

**DOI:** 10.1155/2022/9659871

**Published:** 2022-01-28

**Authors:** Onalethata Lesetedi, Jose Gaby Tshikuka, Shimeles G Hamda, Mgaywa Gilbert Mjungu Damas Magafu, Roy Tapera, Tiny Masupe, Julius Chacha Mwita

**Affiliations:** ^1^Department of Family Medicine and Public Health, Faculty of Medicine, University of Botswana, Gaborone, Botswana; ^2^Department of Nursing Science, Faculty of Health Sciences, National Pedagogical University, Kinshasa I, Democratic Republic of the Congo; ^3^Department of Public Health, Faculty of Medicine, University of Mbujimayi, Mbujimayi, Eastern Kasai Province, Democratic Republic of the Congo; ^4^Malaria Vaccine Implementation Program (MVIP), OMS/WHO Regional Office for Africa, Cite du Djoue, Boite Postale 06, Brazzaville, Congo; ^5^School of Public Health, Faculty of Health Sciences, University of Botswana, Private Bag 0022, Gaborone, Botswana; ^6^Department of Internal Medicine, Faculty of Medicine, University of Botswana, Gaborone, Botswana

## Abstract

**Background:**

Child mortality is a core indicator for child health and wellness. Botswana reported an under-five-year-old children (UFC) mortality rate of 48 deaths per 1000 live births in 2017 against 152 deaths per 1000 live births in 1971. This was a commendable accomplishment. However, given the current country situation whereby 23% of children are born to women living with HIV, the incidence of mortality among UFC born to women living with and not living with HIV and their survival are better health metrics to inform decision making. Nevertheless, such data are still very scarce in Botswana. The study's objective was to estimate the incidence of UFC mortality among children born to women living with and not living with HIV and to compare UFC survival between the two groups.

**Methods:**

A retrospective cohort study of mortality among UFC was conducted in Botswana, including all UFC born between January 2014 and June 2018. Data were extracted from the National Under-Five Mortality Audit Committee (NUFMAC) database using a standardized data collection tool. The incidence rate of UFC death was estimated as a function of the duration from birth to death. Survival functions of UFC born to women living with and not living with HIV were plotted and compared using Kaplan–Meier survival analysis.

**Results:**

The overall incidence of UFC death was 4.63/1000 child months (CM) (95% CI 4.36–4.90). The incidence of UFC death among children born to women living with HIV was 6.96/1000 CM (95% CI 6.47–7.45) and that of UFC born to women not living with HIV was 4.34/1000 CM (95% CI 4.03–4.65). The overall average and standard error (SE) time to event/death for UFC born to women living with and not living with HIV was 54.80 (0.18) months. The mean (SE) time to death for UFC born to women living with HIV was 52.79 (0.41) months and that of UFC born to women not living with HIV was 55.44 (0.19) months (log-rank *X*^2^ = 37.59, *p* < 0.001). Prematurity or low birth weight was the leading cause of UFC death in both groups; but, it was higher in UFC born to women not living with HIV subgroup than their counterparts. Four cases only or 0.5% of the 806 death cases reported by reporting physicians were attributable to HIV-related complications.

**Conclusion:**

Despite the commendable efforts made in reducing UFC death, the incidence of UFC death among UFC born to women living with HIV in Botswana is still higher, and their survival is shorter compared to UFC born to women not living with HIV. Child survival interventions should prioritize UFC born to women living with HIV to improve their survival.

## 1. Background

Child mortality is a core indicator of child health and well-being. According to the World Health Organization (WHO), the under-five-year-old children mortality rate (U5MR) has decreased by 58%, from an estimated rate of 93 deaths per 1000 live births in 1990 to 39 deaths per 1000 live births in 2017 globally [1]. However, in sub-Saharan Africa (SSA), child mortality still remains a significant public health problem with the U5MR at 76 deaths per 1,000 live births in 2017 [2]. This translates to 1 child in 13 dying before his/her fifth birthday, which is 14 times higher than the average ratio of 1 in 185 in high-income countries and 20 times higher than 1 in 263 in the region of Australia and New Zealand [2].

Human immunodeficiency virus (HIV) infection affects child mortality directly through vertical transmission and indirectly through HIV associated maternal death and/or ill health/psychosocial status [3]. In 2013, 2% of global mortality among under-five-year-old children (UFC) was attributed to HIV infection [4]. However, the successful roll-out of the program for the vertical transmission prevention of HIV has led to fewer infants and children becoming infected with the virus each year [4–7].

Botswana, a country in SSA, is characterized by a high U5MR of 48 per 1000 live births in 2017 [8]. Over the years, Botswana has made significant progress. According to the 2017 Botswana Demographic Survey (BDS), UFC mortality declined from 152 deaths per 1000 live births in 1971 to 48 deaths per 1000 live births in 2017 [8]. While infant mortality declined from 97 deaths per 1000 live births to 38 deaths per 1000 live births, respectively [8]. The decline in child mortality in the mid-2000s was partly due to the introduction of antiretroviral therapy (ART) and high coverage of vertical transmission prevention program [8]. The 2015 Botswana Millennium Goals Report indicates that the country attained the Millennium Development Goal 4 (MDG-4) [9]. However, the U5MR in most health facilities is still very high, and about 40–50% of this mortality is due to neonatal deaths [10]. In its efforts to alleviate the situation, the government of Botswana, in collaboration with its partners, has embarked on several programs to promote the child's health and survival. These efforts include implementing the Integrated Management of Childhood Illnesses (IMCI) strategy in 1998, the introduction of the Accelerated Child Survival and Development Strategy in 2009, a robust routine immunization program, and periodic prevention campaigns (vitamin A supplementation and deworming campaign) as well as vertical transmission prevention program. These initiatives have resulted in a tremendous decline in infant and child mortality and improved maternal health as observed by Statistics Botswana between 2008 and 2013 [11].

As a signatory to the Sustainable Development Goals (SGD), Botswana has a target to reduce U5MR by 25 per 1000 live births. Looking at health metrics from Statistics Botswana [8], there is no doubt that the country will meet the SGD goal. The time frame to attain the SGD goal is, however, not clear as the death and survival details of UFC born to women living with HIV are scanty. This situation may obscure differences or inequalities in the distribution of health outcomes among UFC subgroups and likely mislead policy and interventions. Metrics that provide information on the distribution of health outcomes among UFC subgroups are helpful in policy and decision making because 23% of UFC in Botswana are born to women living with HIV [12]. Therefore, metrics such as the incidence of mortality among UFC born to women living with HIV, survival, and time-to-event (death) are critical when planning and implementing health programs; literature search yields very little on these metrics [8–11], yet this information is key to informing decision making. The current study aimed to (i) estimate the incidence of under-five mortality among UFC born to women living with and not living with HIV, (ii) compare the survival of UFC born to women living with HIV with the survival of those born to women not living with HIV, and (iii) assess the time to event/death in these subgroups.

## 2. Subjects and Methods

### 2.1. Study Design and Area

We undertook a retrospective cohort study of mortality among UFC in districts in Botswana between January 2014 and December 2018.

Botswana has 2 cities, namely, Gaborone, the capital city, and Francistown, and 5 towns, namely, Jwaneng, Orapa, Lobatse, Selibe-Phikwe, and Sowa [13]. The total population of Botswana is 2,024,904 [14] and that of children under five years (UFC) is 237,387 or 12% of the total population. The study population included all UFC born in Botswana between January 2014 and June 2018. We excluded from the study all UFC with unknown place of birth or born outside Botswana, multiple births, and those with undocumented cause of death.

### 2.2. Sample Size, Data Collection, and Assessment

The sample size was exhaustive and included everyone as this was a nationwide study. Data was manually extracted from the National Under Five Mortality Audit Committee (NUFMAC) database and exported into a Microsoft Excel spreadsheet for cleaning and screening.

Extracted data included the following variables: The medical record number of the UFC, sociodemographic and biomedical data: gender, date of birth (DOB), gestational age, birth weight, mode of delivery (spontaneous vaginal delivery (SVD) or caesarean section), maternal HIV status, whether HIV prophylaxis was given, feeding status (exclusive breastfeeding/exclusive formula feeding/mixed feeding), immunizations received by a child, mother's residential area, diagnosis or cause of death including UFC HIV status (as diagnosed by the reporting physician), and date of death (DOD). Aside from exclusion factors mentioned in previous paragraph, UFC missing any of these variables were excluded from the study. Children born in Botswana's health facilities are all scheduled for postnatal care for mothers and newborns based on WHO 2013 guidelines [9]. However, whether or not the numbers of follow-up visits between women not living or living with HIV were comparable was not recorded. Mortality was ascertained as by the reporting physician. UFC who died out of health facilities were excluded from the study.

Data analysis was performed using IBM SPSS version 21 (Chicago, IL). The frequency distribution of sociodemographic and biomedical characteristics known as potential risk factors of UFC death was assessed. They included gender, maternal HIV status, and age at death as a continuous-time variable. Age groups were 0–0.91 months (neonate), 0.92–12.00 months (infant), and 12.01–59.99 months. Age at death was estimated by subtracting the DOB from the DOD of the UFC. Proportions of UFC born to women living with HIV and to women not living with HIV were computed. In addition, the proportions of UFC death by cause as diagnosed by the reporting physician were estimated.

Groups or subgroups were compared using chi-square analysis for categorical data and *t*-student test for continuous data with the level of statistical significance set at *p* < 0.05.

The incidence rate of UFC death was estimated as a function of the duration from birth to death. To compute the incidence of deaths among UFC, the rate of new deaths was calculated as the number of new deaths divided by the total child months of follow-up (CM). The CM estimated the actual time-at-risk in the months that the UFC contributed to the study. UFC death being the event/status, survival was estimated as the time elapsed from DOB to the DOD of the UFC or the end of the study. Cases which were lost to follow-up were censored at the last time they were seen while those who stayed until the end of the study without dying from any conditions were censored at the end of the study. To investigate whether survival was the same between UFC born to women living with and not living with HIV, Kaplan–Meier survival analysis was performed. The average time-to-event/death was estimated for UFC born to women living with HIV and for UFC born to women not living with HIV and their 95% confidence intervals (CIs) were computed. The survival function was plotted for the UFC born to women living with HIV and for the UFC born to women not living with HIV and we were checked whether they were similar using the log-rank (Mantel–Cox) chi-square.

### 2.3. Ethical Approval

Ethical approval was obtained from the University of Botswana Institutional Review Board and the Health Research Development Committee (HRDC) of the Ministry of Health and Wellness in Botswana. Permission to access UFC records was sought and obtained from the Child Health Department of the Ministry of Health and Wellness. Consent to participate was not required as this was a record-based study.

## 3. Results

Overall, 12,798 UFC were investigated. Data presented in Table 1 show sociodemographic and biomedical characteristics of UFC born to women living with and not living with HIV between January 2014 and December 2018. Of the 12,798 UFC investigated, 7,264 (56.8%) were male. The median age (IQR) of participants was 8.5 (0.6–22.1) months; that of UFC born to mothers living with HIV was 7.4 (0.7–11.1) months and for UFC born to mothers not living with HIV was 9.2 (0.5–23.2) months. The youngest UFC was 0.1 months while the oldest was 59.74 months. There were 5453 UFC (42.6%) in the age group of 12.01–59.9 months. UFC born to women living with HIV numbered 3300 or 25.8% of the study population, including 10 (0.3%) who were infected through vertical transmission. Under-five-year-old children born to women not living with HIV totalled 9,498; none of them was HIV positive.

The overall follow-up time was 174,165 child months (CM), the total number of events or deaths was 806 (including 4 HIV-related deaths), corresponding to an incidence of 4.63/1,000 CM (95% CI 4.36–4.90). The total CM follow-up among UFC born to women living with HIV was 40,382.73 and the total number of events or deaths was 281, corresponding to an incidence of 6.96 CM (95% CI 6.47–7.45). The total CM follow-up among UFC born to women not living with HIV was 133,782.32 and the total number of events/deaths was 525, corresponding to an incidence of 4.34/1,000 CM (95% CI 4.03–4.65).

Leading causes of death as by the reporting physician among UFC born to women living with and not living with HIV are given in Table 2. Of the 806 death cases recorded among UFC, 359 or 44.5% were caused by prematurity/low birth weight. Other causes were birth asphyxia, diarrhoea, septicaemia, pneumonia, and malnutrition. There were 4 (0.5%) cases of HIV-related UFC death.

Significant (*p* < 0.05) differences between UFC born to women living with and not living with HIV were noted in mortality, mainly attributable to prematurity/low birth weight, pneumonia, and malnutrition. The cause of death was unknown for 12.8% of UFC born women living with HIV and 20.0% of UFC born to women not living with HIV (*p* < 0.001). UFC deaths were comparable between the two groups for the rest of the leading causes of mortality.

Results presented in Table 3 show the distribution of deaths by age among UFC born to women living with and not living with HIV. Seventy percent (70%) of deaths occurred among UFC aged between 0.0 and 91.00 months, 24% among UFC age between 0.92 months and 12.00 months while 9.00% of death occurred among UFC aged more than 12 months.

The average time-to-event for UFC born to women living with and not living with HIV is presented in Table 4. The mean and (SE) time-to-event/death for the overall UFC population was 54.80 (0.18) months (95% CI 54.46–55.15). The mean and (SE) time-to-event for UFC born with women living with HIV was 52.79 (0.41) months (95% CI 51.98–53.59) and for UFC born to women not living with HIV, it was 55.44 (0.19) months (95% CI 55.07–55.82). The log-rank (Mantel–Cox) chi-square showed significant (*p* < 0.001) survival difference between the subgroups.

**Figure 1:**
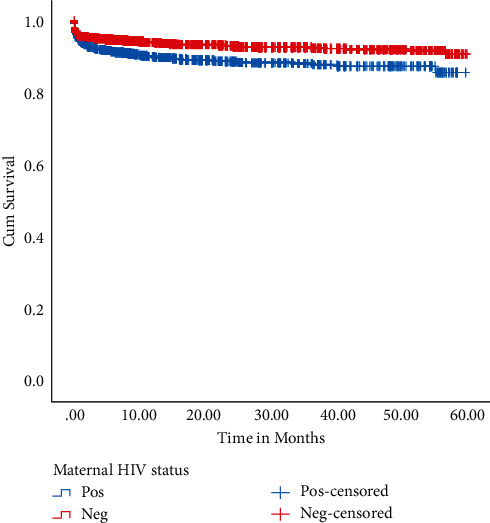
Kaplan–Meier survival curves of UFC born to women living with and not living with HIV between Jan 2014 and Dec 2018 in Botswana (*N* = *n* = 12,798) (Log-rank (Mantel–Cox) chi-square = 37.59, *p*=0.001).

Kaplan–Meier survival curve in Figure 1 showed a significant (*p* < 0.001) difference between UFC death for UFC born to women living with HIV and UFC death for UFC born to women not living with HIV. 3019 (91.5%) UFC born to women living with HIV were censored, while 8973 (94.5%) UFC born to women not living with HIV were censored.

## 4. Discussion

This study found out that only 0.3% of UFC born to women living with HIV were HIV positive by the reporting physician's diagnosis. The incidence of UFC death was significantly higher in this subgroup compared to UFC born to women not living with HIV. Their survival and average time to death were also shorter compared to their counterparts born to women not living with HIV. The finding suggests that mortality of under-five-year-old children born to women living with HIV was not associated with the UFC serological status but with factors related to their mother serological status.

Studies supporting these findings are in the literature [3, 15–21]. Of particular interest is the study conducted by Zash and coworkers in Botswana in 2016. These investigators (Zash et al.) recruited mothers and their newborns directly from postpartum wards in 5 different Botswana communities and followed them up for 24 months. They estimated the cumulative incidence of UFC death per 1000 child-years and compared outcomes between UFC born to women living with HIV and UFC born to women not living with HIV. Their results showed profiles very comparable to the findings presented herein. UFC born to women living with HIV had a higher incidence of mortality and shorter survival and time-to-event than their counterparts.

Similar results have also been reported in neighbouring Zimbabwe where noninfected UFC born to women living with HIV had a death incidence of 150 per 1000 person-years compared to their counterparts born to women not living with HIV who had a death incidence of only 47 per 1000 person-years [3]. Outside the subregion, comparable results have also been reported [3, 15, 22, 23]. What comes out of all these studies is that there are factors associated with mothers that independently affect UFC death and that strategies currently seeking improvement of UFC death are not fully addressing. Intervention programs are needed to get UFC death comparable between the 2 subgroups.

The use of secondary data in this study is a limiting factor, since we were unable to analyse variables other than those collected by the primary users. Also, the extensive exclusion of potential participants due to missing data and loss to follow-up bias to which retrospective studies are prone are other limitations of this study. Further studies using primary data may help to establish better facts brought up by this study. However, using a nationwide dataset of 12,798 persons and 5 years' follow-up gives strength and inferentiality to the outcomes.

While it is difficult to imagine how the investigator or participant could be influenced by awareness of an outcome that has not yet occurred in prospective cohort studies, in retrospective cohort studies participants generally know both their exposure status and the outcome. Thus, these studies are prone to selection bias. Subjects with both the exposure and the condition under study are more likely to be attracted by the study than those without, especially if questions under study have some potential liability or monetary consequences tied to the outcome. This may of course cause differential retention of subjects in relation to the exposure and the outcome, which will lead to an overestimation or underestimation of the association or loss to follow-up bias. This might add up to limiting factors of this work.

However, in this particular study, the likelihood of this bias was very low as no contact was made with subjects. Variables and questions addressed herein are those routinely collected and reported by reporting physicians during postnatal follow-up care. No potential liability or monetary consequences are tied to the outcome of the follow-up. Also driven phenomenon rather than anything else. After delivery every mother is excited to know where her child is keeping up with the recommended WHO growth curve [9] and, wants to make sure that her child receives all recommended vaccines in time. This is perhaps what best explains the number of their contacts with the health system rather than their serological status.

In this study, all the women living with HIV were on ART and enjoyed good health status. Only 0.3% of their offspring had HIV infection through vertical transmission; it is therefore difficult that ascertainment of death among children born to women living with HIV be explained by the number of their contacts with the health system.

The study brings in new inputs that may foster strategies aiming to improve UFC health and wellness. Differences observed between UFC subgroups may call for subgroup tailor-made public health actions to improve UFC health and wellness in general.

## Figures and Tables

**Table 1:** Sociodemographic and biomedical characteristics of under-five children born to women living with and not living with HIV between January 2014 and December 2018 in Botswana (*N* = 12,798).

Characteristic	Mother living with HIV		Mother not living with HIV		*p* value
	Number	Proportion (%)	Number	Proportion (%)	
UFC gender					
** **Male	1846	55.9	5418	57.0	0.270
** **Female	1454	44.1	4080	43.0	
** **Total	3300	100	9498	100	
Age median (IQR)	7.4 (0.7–11.1)	—	9.2.(0.5–23.2)	—	
UFC age group (months)					
** **0–0.91	951	28.8	2910	30.6	0.001^*∗∗*^
** **0.92–12	1079	32.7	2405	25.3	
** **12.01–59.9	1270	38.5	4183	44.0	
** **Total	3300	100	9498	100	
Mode of delivery					
** **SVD	2676	81.1	7509	79.1	0.013^*∗*^
** **C/S	624	18.9	1989	20.9	
** **Total	3300	100	9498	100	
UFC BW (grams)					
** **<1000	89	2.7	209	2.2	0.001^*∗∗*^
** **1000–1499	224	6.8	561	5.9	
** **1500–2499	737	22.3	1457	15.3	
** **2500–3999	2182	66.1	6893	72.6	
** **>4000	68	2.1	378	4.0	
** **Total	3300	100	9498	100	
UFC gestational age (weeks)					
** **<28	88	2.7	218	2.3	0.001^*∗∗*^
** **28–30	193	5.8	432	4.5	
** **31–33	203	6.1	503	5.3	
** **34–36	524	15.9	1040	10.9	
** **37–41	2233	67.7	7017	73.9	
** **>41	59	1.8	288	3.0	
** **Total	3300	100	9498	100	
HIV prophylaxis at birth					
** **Yes	3207	92.2	—	—	—
** **No	93	2.8			
** **Total	3300	100	9498	100	

Legend: UFC = under-five child, M = mean, SE = standard error of the mean, SVD = spontaneous vaginal delivery, C/S = Caesarean section, BW = birth weight, EBF = exclusive breastfeeding, and EFF = exclusive formula feeding. Comparisons are for within subgroup: ^*∗*^*p* < 0.05; ^*∗∗*^*p* < 0.001.

**Table 2:** Cause of death by the reporting physician among under-five children born to women living with and not living with HIV between January 2014 and December 2018 in Botswana (*n* = 806).

UFC	Overall	Mother status				*p* value
Cause of death	*N* (%)	Mother living with HIV		Mother not living with HIV		
Number	Proportion (%)	Number	Proportion (%)			
Prematurity LBW	359 (44.5)	118	42.0^a^	241	45.9^b^	0.001^*∗∗*^
Birth asphyxia	81 (10.0)	29	10.3	52	9.9	0.91
Diarrhoea	75 (9.3)	35	12.5	40	7.6	0.67
Septicaemia	64 (7.9)	26	9.3	38	7.2	0.45
Pneumonia	49 (6.1)	23	8.2^a^	26	5.0^b^	0.03^*∗*^
Malnutrition	33 (4.1)	10	3.6^a^	23	4.4^b^	0.001^*∗∗*^
HIV/AIDS	4 (0.5)	4	1.4	—	—	—
Unknown/minor condition	141 (17.5)	36	12.8^a^	105	20.0^b^	0.001^*∗∗*^
**Total**	806 (100)	281	100	525	100	

UFC = under-five children, LBW = low birth weight, and ^*∗*^*p* < 0.05; ^*∗∗*^*p* < 0.001; different letters between rows reflect significant differences (*p* < 0.05).

**Table 3:** Distribution of event or mortality by age among under-five-year-old children born to women living with and not living with HIV between January 2014 and December 2018 in Botswana (*n* = 806).

Age (months)	N	Percentage	*p* value
0.0–0.91	540	66.9^a^	0.001
0.92–12.00	193	23.9^b^	0.001
12.1–59.99	73	9.1^c^	0.001
Total	806	100.0	-

*N* = number of under-five-year-old children born to women living with and not living with HIV; different letters within a column reflect significant differences (*p* < 0.05).

**Table 4:** Average time-to-event^*∗*^ for under-five-year-old children born to women living with and not living with HIV between January 2014 and December 2018 in Botswana (*n* = 12,798).

UFC characteristics	Mean survival time or average time-to-the event (months)			
	Estimate	SEM	95% confidence interval	
			Lower limit	Upper limit
UFC born to women living with HIV	52.8	0.4	52.0	53.6
UFC born to women living with HIV	55.4	0.2	55.1	55.8
Average	54.8	0.2	54.5	55.2

^*∗*^Death, log-rank (Mantel–Cox) chi-square = 37.59; df = 1; *p*=0.001; UFC = under-five-year-old children; SEM = standard error of the mean.

## Data Availability

Data underlying the findings in this study are not publicly available to maintain patient confidentiality. The data include potentially identified demographic and clinical care information. However, the data can be requested from the corresponding author, who must first get permission from the Ministry of Health and Wellness and the National Under-Five Mortality Audit Committee before sharing.
